# Manual route modification using an oblique method following automatic virtual bronchoscopic navigation

**DOI:** 10.1097/MD.0000000000029076

**Published:** 2022-05-06

**Authors:** Takako Inoue, Takahisa Kawamura, Kei Kunimasa, Motohiro Tamiya, Hanako Kuhara, Kazumi Nishino, Satomi Odani, Fumio Imamura, Toru Kumagai, Kotaro Miyake

**Affiliations:** a Department of Thoracic Oncology, Osaka International Cancer Institute, 3-1-69 Otemae, Chuo-ku, Osaka, Japan; b Cancer Control Center, Department of Oncology, Osaka International Cancer Institute, Osaka University Graduate School of Medicine, Japan; c Department of Respiratory Medicine and Clinical Immunology, Osaka University Graduate School of Medicine, Japan.

**Keywords:** manual modification, oblique method, peripheral pulmonary lesions, virtual automatic bronchoscopic navigation

## Abstract

Virtual automatic bronchoscopic navigation (VBN) systems to determine the route to peripheral pulmonary lesions (PPLs) in lung cancer can improve diagnostic biopsy yields. However, compared with VBN, drawing manual routes using computed tomography images, especially with oblique methods, can identify more routes. The Ziostation2 VBN system combines the benefits of these 2 methods; we evaluated this performance by comparing 3 different route-determining methods.

We retrospectively collected data from 50 patients with PPLs measuring <30 mm who underwent transbronchial biopsy with an ultrathin bronchoscope at the Osaka International Cancer Institute during January to December 2018. We compared automatic VBN (Ziostation2), manual route modification using an oblique method after automatic VBN, and manual navigation using a general application computed tomography viewer. Concordance between predicted and actual branching were determined. We also compared the predicted relationship between the terminal bronchi and the lesion by 2 of the methods with ultrasonographic images (radial-probe endobronchial ultrasonography [radial-EBUS]).

Manual modification after automatic VBN significantly increased the rate of determining routes to the target (66%) versus with the automatic VBN alone (32%) (*P* < .001). Expected route bifurcations were exact matches with actual branching in 45/48 of the patients using manual modification after automatic VBN. The predicted relationship between the terminal bronchi and the lesion using manual modification after VBN matched the radial-EBUS images in 35/50 of the patients.

Manual modification of routes to PPLs using an oblique method after automatic VBN predicted actual radial-EBUS route imaging and could help determine appropriate patients for bronchoscopy.

## 1 Introduction

With recent advances in computed tomography (CT) for evaluating lung cancer, the detection size of pulmonary peripheral lesions has decreased, and we can detect fainter lesions. In addition to surgical resection, the treatment options for early lung cancer, such as stereotactic radiotherapy and heavy particle radiotherapy, are varied, and the demand for a definitive diagnosis of early lung cancer without a surgical procedure is increasing.^[1]^

Various approaches are available for diagnosis when performing biopsies of these suspicious lesions. Recent modifications to bronchoscopy, such as the use of radial-probe endobronchial ultrasonography (radial-EBUS) with or without a guide sheath (GS), and virtual bronchoscopic navigation (VBN) have increased the diagnostic yield of bronchoscopy for small peripheral lung lesions.^[2]^ These techniques are recommended by the American College of Chest Physicians lung cancer guidelines.^[3]^ With VBN, the diagnostic yield is reportedly superior to that with manual bronchoscopic navigation (MBN) involving a manual analysis of 2-dimensional CT images. Bronchoscopic guidance lets physicians select the route to the target, and the importance of selecting a bronchus under bronchoscopic direct vision has increased compared with the importance of adjustment under fluoroscopic imaging.^[2,4]^ Therefore, ultrathin bronchoscopy (UTB) is expected to be effective. Additionally, in one study, the diagnostic yield of the novel 3.0-mm ultrathin bronchoscope directed by radial-EBUS, VBN, and fluoroscopy was higher than that of conventional 4.0-mm thin bronchoscopes directed by the same methods.^[5]^ However, thinner bronchoscopes require more accurate navigation because these are able to reach more distal bronchi. In addition, the bronchoscopic instruments in UTB for sampling are smaller compared with the conventional procedure, and the sampling area acquired by UTB is narrower. Inadequate navigation is directly involved in biopsy failure.

The direct oblique method (DOM) is a MBN technique that is not a software program but rather a manual procedure conducted using a general application CT viewer.^[6]^ DOM supports precisely identifying airways without mental reconstruction, and the accuracy of identification and bronchi selection both in the drawing phase and in the bronchoscopic simulation phase is reportedly better than with automatic VBN.

It is important to develop a bronchoscopic guidance system that supports physicians by providing greater accuracy and visualization of images of the bronchial bifurcations than existing VBN systems. Some existing VBN systems, namely VINCENT-sim (Fujifilm Medical, Tokyo, Japan), DirectPath (Olympus Medical Systems, Tokyo, Japan), and Bf-NAVI (Olympus Medical Systems) can be used with manual CT analysis. With these systems, for example, DirectPath and Bf-Navi, it is possible to create a virtual image in the vicinity of the lesions by additional extraction if the automatic extraction of the bronchi is insufficient. Completing a bronchial tree by placing dots in the bronchi on the CT images from the target to the terminal of the automatic extraction can confirm a virtual bronchus. Although this option is especially useful, and the developers also recommend manual additional extraction, it is necessary to draw the bronchi three-dimensionally with CT, which requires time. Therefore, techniques for tracking branches three-dimensionally are needed because the CT images browsed for additional bronchi are not cross- or vertical sections of the bronchi.

For these reasons, we considered it important for navigation to make manual modifications of bronchi easier and more accurate. Additional extraction using the oblique method makes it easy to confirm the continuity of the bronchi because it is possible to track the bronchi using cross- and vertical sections that rotate freely around the route as an axis. Because VB-like cross-section oblique CT images of the route can be used in the simulation phase, route addition is possible simply by adding dots on the CT images as a marker for each route branch. In this study, we investigated the usefulness of a method to perform additional extraction using the oblique method with automatic bronchial extraction obtained by VBN.

## 2 Materials and methods

This study was approved by the institutional review board of our institute.

This study was observation study designed to evaluate the accuracy of the manual modification by the oblique method with the Ziostation2 compare with automatic VBN to that of automatic VBN and that of direct oblique method.

### 2.1 Study subjects

We retrospectively collected data from all patients with small peripheral pulmonary lesions (PPLs) (<30 mm on axial CT images) who underwent bronchoscopic examination with a 3.0-mm ultrathin bronchoscope (BF-MP290F; Olympus Medial Systems) at the Osaka International Cancer Institute, Japan, during January to December 2018. A total of 50 subjects were included in this study. Data comprised thin-section CT images and the calculated routes by automatic VBN (Ziostation2 CT bronchoscopic navigation, hereafter referred to as “Ziostation2”; Ziosoft, Tokyo, Japan) described below as automatic VBN (Ziostation2 CT bronchoscopic navigation, hereafter referred to as “Ziostation2, Zio(A)), with modification using the oblique method with the Ziostation2 automatic VBN (described below as modification using the oblique method with the Ziostation2 automatic VBN, Zio(M)) and with DOM using a general application CT viewer (aquarius thin-client viewer, hereafter referred to as “Aquarius”; Tera Recon, San Mateo, CA).

All patients underwent bronchoscopic examination directed by 2 navigation systems (Ziostation2 and DOM using Aquarius), radial-EBUS, fluoroscopy, and rapid onsite evaluation.

### 2.2 CT data for the navigations

First, CT was performed using a helical CT scanner (Aquilion ONE and Aquilion PRIME; Canon Medical Systems Corporation, Tochigi, Japan). The bronchus involved in the lesion was evaluated on images obtained by reconstruction at ≤0.65-mm intervals using the chest algorithm (window width: 1500 Hounsfield units (HU), window level: −600 HU) from helical CT data. Although reconstruction at ≤0.65 mm is recommended, this is unnecessary for analysis using DOM and VBN in Ziostation2.

### 2.3 DOM

DOM is not a software program, but a manual procedure conducted using general application CT viewers. DOM can be analyzed in general application CT viewers with availability of oblique CT images and manual placement of dots on the CT images. DOM can be conducted using several CT viewers, namely Vincent (Fujifilm Medical), the Aquarius thin-client viewer, Ziostation, and OsiriX.^[6]^

The concept of DOM is that adequately adjusted two-dimensional oblique CT images are VB-like regarding the shapes and angles of bronchial bifurcations, which is necessary and sufficient for performing bronchoscopy. Compared with traditional MBN, DOM does not require mental reconstruction in which the physician mentally visualizes a complex three-dimensional airway construction by scrolling through contiguous 2-dimentional CT images. In this study, the DOM procedure was implemented as follows using the Aquarius thin-client viewer: in the drawing phase, a bronchus leading to the target was manually identified and manually marked with dots on the CT images. The bronchus was confirmed using cross-sectional CT.

### 2.4 VBN manual modification using DOM

**Figure 1:**
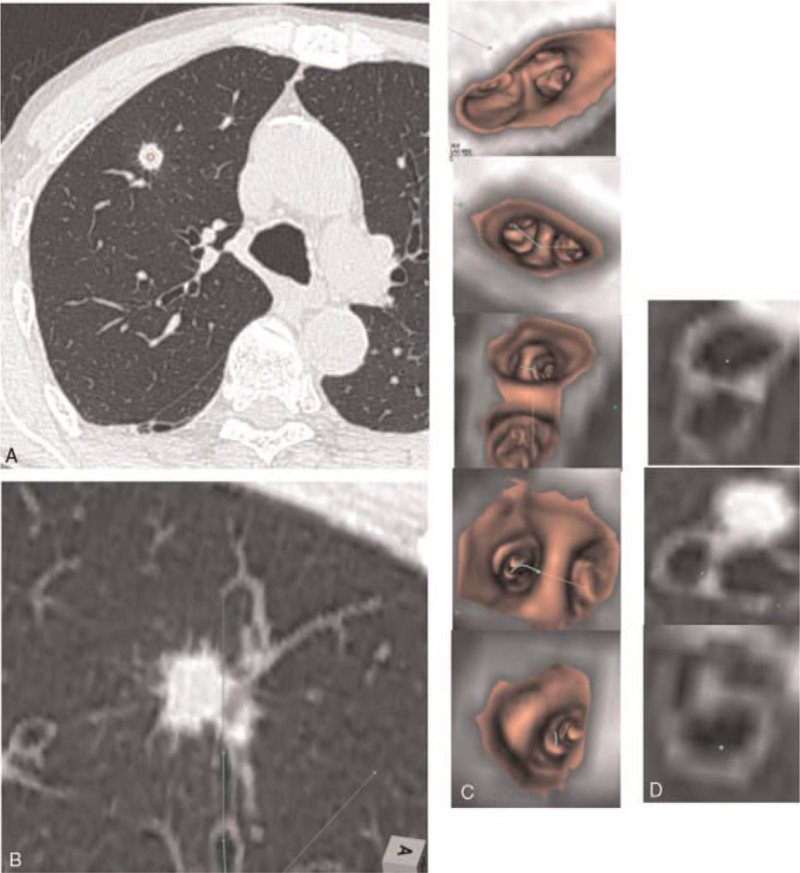
(A) No bronchus sign is seen using axial computed tomography (CT). (B) Image modification using the direct oblique method on CT images to draw the route to the target. (C) As for conventional virtual bronchoscopic (VB) navigation systems, VB images of each bronchial branching on the route to the lesion are shown as an animated image, and the bronchi for bronchoscope advancement are marked. (D) To confirm the peripheral bronchi from the limit of the VB image animation, the bronchial representation shifts to oblique CT, maintaining the visual axis seamlessly with fusion images created from the VB images and oblique CT images.

The concept of modification using DOM with automatic VBN in Ziostation2 is to connect the adequately adjusted two-dimensional oblique CT images to automatically extracted bronchi seamlessly both in the drawing phase and in the bronchoscopic simulation phase (Fig. 1). In the drawing phase, the system performs automatic airway segmentation to reconstruct the airway tree. The operator defines a target by placing markers on the CT images. Software route calculation shows the candidate routes (mainly 2–3 routes) to the target, and cross-sectional and freely rotating vertical-section two-dimensional oblique CT images of selected routes are shown automatically. The physicians modify the selected routes, placing dots at each bifurcation from the periphery to the center using an adequate oblique CT image. Once the dot by the physician meets the route calculated by the system, the new modified route is generated. The physician can select the best routes using cross-sectional CT images and vertical-section oblique CT images that rotate freely around the bronchial axis and can then estimate the existence of small peripheral bronchi.

In the bronchoscopic simulation phase, the drawn routes are shown by VB. The operator refers to the VB images up to the central bronchus (lobar bronchus to segmented bronchus in most cases), and on the peripheral side, then refers to the corresponding cross-sectional oblique CT images of the marked route. Adequately adjusted two-dimensional oblique CT images are VB-like regarding the shapes and angles of the bronchial bifurcations, which eliminates the need for mental reconstruction.

After switching VB images to oblique CT, the oblique CT accurately provides the necessary and sufficient information, such as branch selection, angle, and the distance required for the bronchoscope. The signpost of the bronchi is also marked on the CT images, and the thumbnail of the VB images and oblique CT images at each bronchial bifurcation is displayed as a catalog.

**Figure 2:**
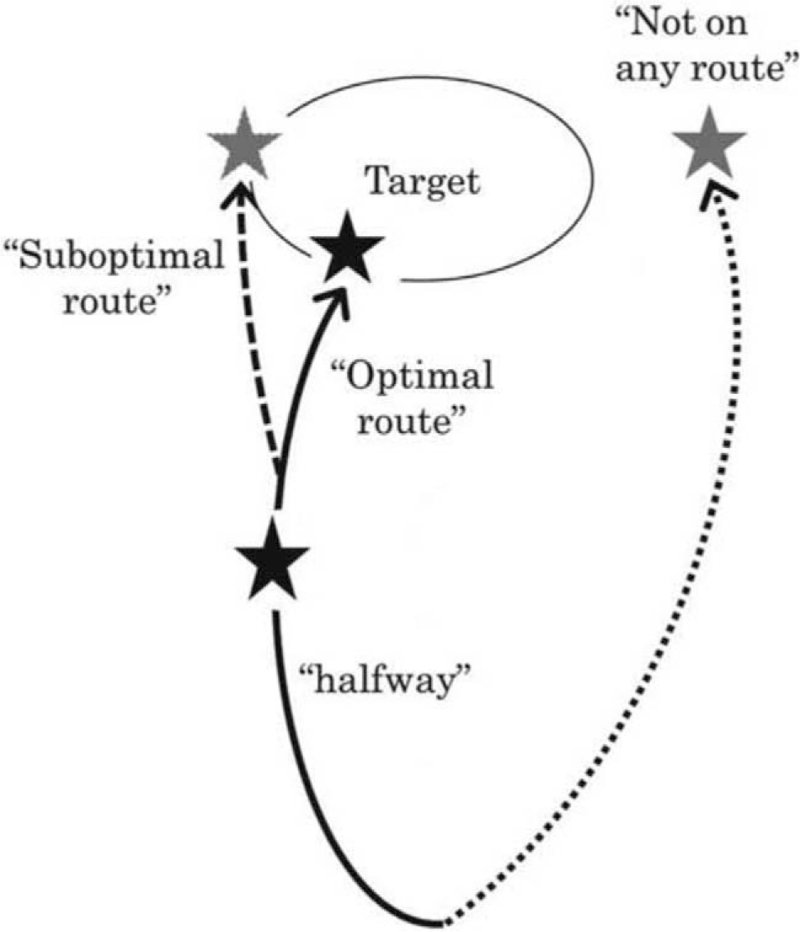
Classification of routes obtained by navigation. Optimal route: terminal route inside the lesion suboptimal route: terminal route is adjacent to the lesion not on the route: not on any route to the target, with no route leading to the target. When ≥1 bifurcation between the terminal bronchi and the lesion was detected by other navigation, we added “halfway” to each classification.

Routes were calculated for the 50 lesions using Ziostation2 and a general application CT viewer (Aquarius). The route was classified as follows (Fig. 2): “optimal route”: terminal route inside the lesion; “suboptimal route”: terminal route is adjacent to the lesion; and “not on the route”: not on any route to the target, with no route leading to the target. When ≥1 bifurcation between the terminal bronchi and the lesion was detected by other navigation, we added “halfway” to each classification.

We verified the automatic bronchoscopic navigation system regarding how many drawn routes led to targets in the drawing phase, and then how additional manual modification improved route selection. The VBN operator then chose the best route among the presented options (2–3 candidates). In 2 cases, airway identification for virtual navigation was difficult using Ziostation2 because the existing lung anatomy was abnormal owing to chronic inflammation and postoperative changes. In these cases, it was possible to confirm the bronchi using DOM.

In order to assess inter-reliabilities coming from different operators, these and other classifications were independently confirmed by 2 respiratory specialists who had been trained in DOM for at least 1 year. At the time of bronchoscopy, both results were discussed, and the best route was chosen for the bronchoscopy.

### 2.5 Statistical analysis

The statistical analyses were performed with EZR, version 1.37 (Saitama Medical Center, Jichi Medical University, Saitama, Japan), which is a graphical user interface for R (The R Foundation for Statistical Computing, Vienna, Austria).

The correlation of the locational relationship between the terminal bronchi and the lesion predicted by 3 navigation methods with ultrasonographic images acquired by radial-EBUS was tested by Spearman rank correlation coefficient, denoted by “rs.” For comparisons of the mean values for each group, we used the chi-square test. All statistical tests were 2-sided; *P* ≤ .05 was considered significant. Odds ratio and 95% confidence intervals were also calculated.

If we were to compare the airway detection rate by the Ziostation2 method (expected to be around 90%) versus that of the conventional automatic VBN (previously reported to be around 70%^[6]^ and applied the statistical power at 0.9 [2-sided]) and significance level at 0.05, a total of 40 patients would be sufficient.

To assess inter coefficients for agreement in the route evaluation results between 2 observers, kappa (*κ*) coefficients were calculated. The *κ* coefficients were calculated using BellCurve for Excel version 3.21 (Social Survey Research Information Co., Ltd.).

## 3 Results

### 3.1 Characteristics of the patients and lesions

We enrolled 50 patients in this study, and the patients’ characteristics are summarized in Table 1. The median lesion size in the longest diameter on axial CT was 18.0 mm (range: 8.0–30.1 mm); 94% (47/50) of the lesions were solid, 62% (31/50) of the lesions measured <20 mm, and 30% (15/50) of the lesions were invisible under fluoroscopic imaging. Bronchus signs on axial CT images were detected in 70% (35/50) of the patients. Among 15 targets for which bronchus signs on axial CT images were undetected, a route leading to within the target was detected for 5 targets (33%), and a route leading adjacent to the target was detected for 4 targets (27%) using DOM. Zio(A) was able to draw routes to within the targets for 3 targets (20%), and Zio(M) was able to draw routes to within the targets for 7 targets (47%).

**Table 1:** Characteristics of the lesions.

Characteristics	N = 50
Lesion size in the longest diameter on computed tomography (CT)	
Median(range), mm	18.0(8.0–30.1)
<20 mm	31(62%)
>20 to <30 mm	19(38%)
Lesion located in bronchopulmonary segment	
Right upper lobe	16(32%)
Right middle lobe	3(6%)
Right lower lobe	10(20%)
Left upper lobe	14(28%)
Lingula	2(4%)
Left lower lobe	5(10%)
Bronchus sign on axial CT	
Present	35 (70%)
Absent	15 (30%)
Appearance on CT	
Solid	47 (94%)
Others	3 (6%)
Fluoroscopy	
Visible	35 (70%)
Invisible	15 (30%)
Final diagnosis	
Malignant	42 (84%)
Benign	7 (14%)
Unknown	1 (2%)

### 3.2 Predictive image of bronchoscopic navigation in the drawing phase

Table 2 shows how the best route to the target was classified in each navigation. DOM drew routes to terminals inside the lesions for 36 (72%) cases, and to terminals adjacent to the lesions for 12 (24%) cases. For 2 cases, the pulmonologist could not recognize any bronchi or artery leading to the lesions even when using both cross-sectional and vertical-section oblique CT images.

**Table 2:** Predictive image of bronchoscopic navigation in the drawing phase.

	Zio (A)	Zio (M)	DOM
Optimal route	16 (32%)	33 (66%)	31 (62%)
Optimal route (halfway)	13 (26%)	3 (6%)	5 (10%)
Suboptimal route	6 (12%)	9 (18%)	10 (20%)
Suboptimal route (halfway)	7 (14%)	2 (4%)	2 (4%)
Not on the route	6 (12%)	1 (2%)	2 (4%)
Not evaluable	2 (4%)	2 (4%)	0 (0%)

DOM = direct oblique method, Zio (A) = Ziostation2 automatic virtual bronchoscopic navigation, Zio (M) = manual modification using the oblique method based on the Ziostation2 automatic virtual bronchoscopic navigation.

**Figure 3:**
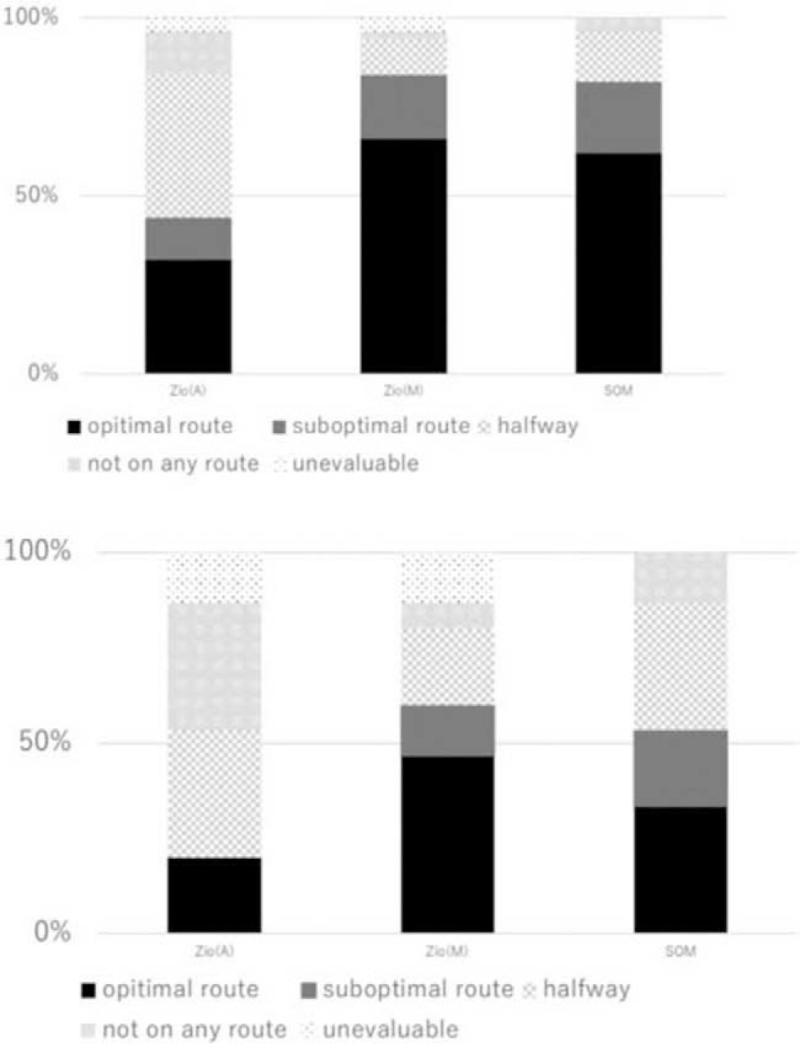
(A) Predictive image of bronchoscopic navigation in the drawing phase. (B) Predictive image of bronchoscopic navigation in the drawing phase in the lesions in which bronchus signs were negative as evaluated by axial CT images. CT = computed tomography.

The best route provided by Zio(A) was classified “optimal route” in 16 cases (32%), “optimal halfway” in 13 cases (26%), “suboptimal route” in 6 cases (12%), “suboptimal halfway” in 7 cases (14%), and “not on the route” in 6 cases (12%). Manual modification by the oblique method increased the rate (34%, 17/50) to calculate the new “optimal route” to the target in addition to the automatic VBN (Fig. 3A). In the lesions in which bronchus signs were negative as evaluated by axial CT images, an inflow route to the target was extracted in 46% (7/15) of the routes by Zio(M) compared with 20% (3/15) by Zio(A) (Fig. 3B). When the routes provided by Zio(A), by Zio(M), and by DOM were independently classified by 2 operators, *κ* coefficient between the 2 operators were, 0.81 (95% confidence interval 0.68–0.94), 0.73 (95% confidence interval 0.56–0.90), 0.86 (95% confidence interval 0.73–0.98), respectively.

### 3.3 Comparison of the prediction by the navigation methods with ultrasonographic images acquired by radial-EBUS

Ultrathin bronchoscopy was performed without complications, and the median examination time was 27 minutes (range, 12–66 minutes). Ultrathin bronchoscopy confirmed directly that the radial-EBUS probe advanced through the final bifurcation to the target in 32 (64%) cases, and in 18 (36%) cases, the ultrathin bronchoscope advanced 1 or 2 branching before the bronchus, for which navigation could be analyzed. For 45 (90%) cases, the bronchial branching predicted by the navigations exactly corresponded to the actual branching. For 2 cases (4%), there was an overlooked fine bronchial branching by all navigation systems when analyzing the CT images. In 1 case (2%), there was more peripheral bronchial branching after the limit of the system's analysis. For 2 cases (2%), we could not confirm the actual branching, retrospectively.

The relationship between the prediction by navigation and ultrasonographic imaging is shown in Table 3. In 77.8% (28/36) of the cases with calculated terminals “within the target” by oblique CT, the ultrasonographic image by radial-EBUS matched the prediction. In 97.2% (35/36) of the cases, we obtained an ultrasonographic image (within the lesion, adjacent to the lesion).

**Table 3:** Comparison of the route prediction by the navigation methods with ultrasonographic images acquired by radial-EBUS.

Zio automatic				
	Within	Adjacent to	Invisible	Total
Optimal route	22	6	1	29
Suboptimal route	5	6	2	13
Not on the route	3	1	2	6
Not evaluable	0	2	0	2
Total	30	15	5	50

Zio modified by oblique method				
	Within	Adjacent to	Invisible	Total
Optimal route	28	7	1	36
Suboptimal route	2	5	3	10
Not on the route	0	1	1	2
Not evaluable	0	2	0	2
Total	30	15	5	50

Zio = Ziostation2 computed tomographic bronchoscopic navigation.

The prediction by Zio(M) showed stronger correlation with the ultrasonographic image by radial-EBUS (rs = 0.606, *P* < .01) than that of Zio(A) (rs = 0.395, *P* < .01).

### 3.4 Diagnostic yield

The diagnostic yields in histologic specimens and the factors affecting the diagnostic yields are shown in Table 4. Larger PPLs (≥20 mm) had a tendency to be associated with a higher diagnostic yield than smaller PPLs (<20 mm). Lesions with a positive bronchus sign on axial CT images had a tendency to be associated with higher diagnostic yield than when the bronchus sign was absent in PPLs on axial CT images (11/15 [73.3%] vs 32/35 [91.4%], respectively; *P* = .213). Using the prediction by Zio(A), the diagnostic yields of the cases with calculated terminals “within the target” was significantly higher than the cases with calculated terminals “adjacent to the target” or “not on the route” (28/29 [96.6%] versus 7/11 [66.7%] vs 5/7 [71.4%], respectively; *P* = .046).

**Table 4:** Factors contributing to bronchoscopic diagnosis.

	Positive consultation number of lesions/total lesions (%)	*P* value
Lesion size, mm		
20–30, n (%)	24/31 (77.4%)	
<20, n (%)	19/19 (100%)	.070
Bronchus sign on axial CT		
Present	11/15 (73.3%)	
Absent	32/35 (91.4%)	.213
Analysis by Zio (A)		
Optimal route	28/29 (96.6%)	
Sub-optimal route	8/12 (66.7%)	
Not on the route	5/7 (71.4%)	.046
Analysis by Zio (M)		
Optimal route	34/36 (94.4%)	
Sub-optimal route	7/11 (63.6%)	
Not on the route	0/1 (0%)	.004
The image of radial-EBUS		
Within	30/30 (100%)	
Adjacent to	12/15 (80%)	
Invisible	1/5 (20%)	<.001

CT = computed tomography, radial-EBUS = radial-probe endobronchial ultrasonography, Zio (A) = Ziostation2 computed tomographic bronchoscopic navigation, Zio (M) = Ziostation2 automatic virtual bronchoscopic navigation.

Using the prediction by Zio(M), the diagnostic yields of the cases with calculated terminals “within the target” was significantly higher than for cases with calculated terminals “adjacent to the target” or “not on the route” (34/36 [94.4%] vs 7/11 [63.6%] vs 0/1 [0%], respectively; *P* = .004).

The position of the radial-EBUS probe affected the diagnostic yields. Cases in which the probe was confirmed “within the target” had significantly higher diagnostic yield than cases in which the probe was confirmed “adjacent to the target” (30/30 [100%] vs 12/15 [80%], respectively; *P* < .001).

## 4 Discussion

This was a retrospective study evaluating the new VBN system, Ziostation2, which is based on the oblique method. In this system, we emphasized the ease of manual modification rather than improving the capabilities of automatic analysis. As a result, improvement of a selected route by manual modification was achieved in 22/48 (45.8%) cases, which suggested that fully automatic analysis may be insufficient in nearly half of cases.

We believe that manual modification is necessary for accurate bronchial evaluation. In addition, our results showed no significant difference between the results of fully manual analysis versus manual modification using the oblique method with automatic analysis. Manual modification based on the oblique method with automatic analysis could be useful because it can be used easily without experience or without special techniques. This approach is advantageous in theory; therefore, to verify the usefulness in clinical practice, we compared the navigation results with the results of actual bronchoscopy. Previous studies found that findings using EBUS-GS are causally linked to the diagnostic rate.^[7]^ Therefore, it is meaningful to predict the type of image with EBUS-GS, especially whether the probe is within the lesion. In this respect, the results with Zio(M) predicted 28 of 30 cases (93.3%) which were subsequently confirmed as “within the lesion” using EBUS-GS. Conversely, with Zio(A), it was possible to predict only 22 of 30 cases (73%) subsequently confirmed as “within the lesion” using EBUS-GS. These findings suggest that the probability of the bronchoscope being “within the target” is low by radial-EBUS when the evaluation of the route to the target calculated by Zio(M) is not “optimal” or “optimal halfway.” We believe that evaluating routes on oblique CT images may identify important criteria for selecting appropriate cases for bronchoscopy.

There may be no need to analyze the bronchi in high detail because the range of the bronchoscope reach is limited. Indeed, the rate of arrival at the final bifurcation was 64%, in this study. However, in most of the other cases, the bronchoscope could be advanced one additional branching before reaching the final bifurcation. In some cases, reaching the final bifurcation is critical to collect samples. In addition, evaluating the relationship between the target and the final bronchus is useful to select the bronchoscope and the sampling device. If the device can be advanced to within the target, forceps are the appropriate sampling device, and if the device can be advanced adjacent to the target, transbronchial aspiration cytology (TBAC) and curettage using thin bronchoscopy are options to consider.^[5]^

In this study, the navigation modified by the Ziostation2 could not completely predict the radial-EBUS image. We have 2 hypotheses for this limitation; 1 is the limit of the bronchoscope's advancement. As mentioned above, in only 64% of the cases could we insert the radial-EBUS to the final bronchus under direct view. The radial-EBUS probe is a delicate device; physicians tend to avoid applying strong pressure, and it is sometimes difficult to bend this equipment at a sharp angle. In some cases, the proper material was sampled by forceps despite having an “adjacent to radial-EBUS” image. The other hypothesis is that during bronchoscopy, we sometimes inject a small amount of saline into the peripheral bronchus to clear the view while the bronchoscope is wedged in the bronchus, and the obstructed bronchus under CT scan may dilate when the sampling device is advanced to the target.

In conclusion, modification using the oblique method with VBN systems could predict the ultrasonographic image by radial-EBUS, which could lead to the identification of important criteria for selecting appropriate cases for bronchoscopy.

## Author contributions

**Conceptualization:** Takako Inoue.

**Data curation:** Takako Inoue.

**Formal analysis:** Takako Inoue, Satomi Odani.

**Funding acquisition:** Takako Inoue.

**Investigation:** Takako Inoue, Takahisa Kawamura, Kei Kunimasa, Motohiro Tamiya, Hanako Kuhara, Kazumi Nishino.

**Methodology:** Kotaro Miyake.

**Project administration:** Kotaro Miyake.

**Supervision:** Fumio Imamura, Toru Kumagai, Kotaro Miyake.

**Writing – original draft:** Takako Inoue.

**Writing – review & editing:** Toru Kumagai, Kotaro Miyake.
